# The WoundWand and Its Novel Use in Burn Excision Surgery

**Published:** 2015-01-08

**Authors:** Guang H. Yim, Zeeshan Ahmad, Steven L. A. Jeffery

**Affiliations:** The Healing Foundation for Burns Research, Department of Burns and Plastic Surgery, Queen Elizabeth Hospital, Mindelsohn Way, Birmingham, B15 2WB, UK

**Keywords:** burn, eschar, full thickness, tangential excision, WoundWand

## Abstract

**Objective:** The authors present the novel use of a new generation surgical ablative tool known as the WoundWand. **Methods:** A 66-year-old woman presented with 18% deep-dermal and full-thickness burns affecting the left side of her body including the total left upper limb, abdomen, and left thigh following lighting a cigarette in bed while on home oxygen therapy. She underwent tangential excision of burn using the WoundWand with immediate reconstruction using split skin grafts and topical negative pressure therapy. **Results:** We found that we were able to easily tangentially excise the hard eschar from delicate areas such as the hand and fingers using the WoundWand. **Discussion:** The authors conclude that the novel use of this device particularly in delicate areas such as dorsum of fingers and hand has some advantages over preexisting methods. Further clinical study may add to the surgical armamentarium of the burns surgeon.

There are many surgical tools at the Burns surgeon's disposal. Traditional methods include Watson, Goulian, and Silver knives particularly for use in tangential excision.^1^ Recent advances in technology have given rise to the more hemostasic monopolar cutting and coagulation diathermy systems as well as bipolar diathermy, which are better suited to tangential excision or where minimizing blood loss is imperative.^2^^-^5 Further technological advances have given rise to the advent of other tools such as the harmonic scalpel which has been widely used in surgery, initially in urological surgery^6^ and now widely used in a variety of (plastic) surgical disciplines including aesthetic surgery for rhytidectomy, skin oncology surgery for block dissections, and head and neck surgery for tumor extirpation.^7^^-^10

Monopolar cautery is extremely effective, but it may cause significant collateral tissue damage.^11^ Thermal damage can have deleterious effects on wound healing, safety, and clinical outcomes in general^12^; this is particularly crucial in burns surgery. Due to these factors, carbon dioxide lasers and radiofrequency devices are gaining favor; this is also due to the lesser thermal collateral damage.^13^ There are several published studies which support the use of these devices in routine daily surgical practice.^14^^,^^15^ Each method has its advantages and disadvantages as well as an appreciable learning curve which the surgeon will have to negotiate.

Pivotal to burns and plastic surgery is the concept of innovation and application of new tools and techniques to a variety of clinical problems. This drive to perform an operation safer, faster, or with fewer complications prompts a surgeon to continue to trial new devices. While devices such as Versajet are being used in the field of burn care, it is known that Versajet is less effective at directly removing the thick hard eschar of full-thickness burns.^16^^-^18 The authors present the use of such a new-generation surgical ablative tool known as the WoundWand (ArthroCare Corporation, Austin, Texas) to discuss both advantages and disadvantages, as well as its potential application in burns surgery.

## METHODS/CASE REPORT

The authors present the case of a 66-year-old retired woman, who presented with 18% flame burns. She sustained the burns after her bed sheets caught fire when she was smoking a cigarette while using home oxygen therapy; for chronic obstructive airways disease. Her medical history included a 60 pack-year history of smoking, liver cirrhosis and concurrent malnutrition.

**Figure 1:**
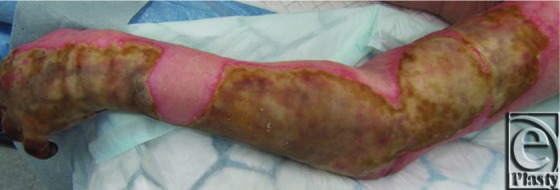
The burn eschar on the left forearm and dorsum of the hand.

**Figure 2:**
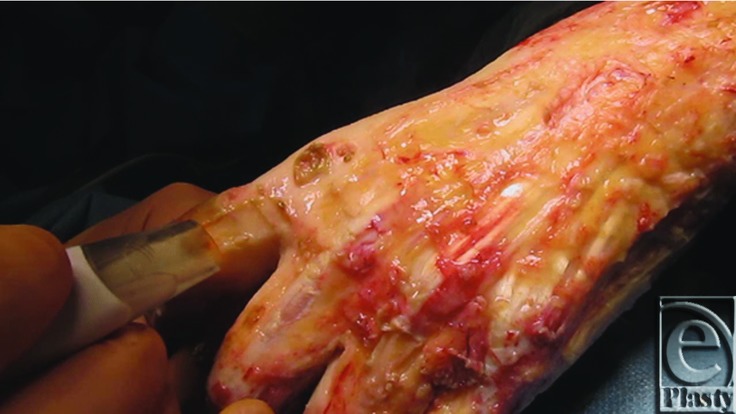
The WoundWand device and excision of eschar from the dorsum of the left index finger.

After detailed assessment, she sustained full-thickness burns to her left thigh, left breast, and left upper limb including her hand, groin, and abdomen (Fig 1). She underwent fascial excision of her burns to these areas. The WoundWand device was used to excise the burns on her arm, hand, and fingers only. On the hand, the paratenon was preserved but burns were deep involving the dorsal skin and subcutaneous tissues (Fig 2). She subsequently underwent meshed skin grafting (1:1 to the hand, 2:1 elsewhere) and had application of topical negative pressure device to all grafted areas, set at 70 mmHg continuous.

A real-time video of the device in use can also be seen at this video link (Video 1. The WoundWand removing eschar from the dorsum of the wrist and left little finger).

When checked 5 days later, she had 50% graft loss to the thigh. The grafts to her left upper limb, breast, and abdomen were intact. Microbiology swabs confirmed presence of coliforms in the burn wounds at initial surgery. This was compounded by low preoperative albumin levels at 16 to 19 g/dL. Unfortunately, at 10 days postoperatively, she had extensive graft loss to her left breast and abdomen requiring regrafting.

**Figure 3:**
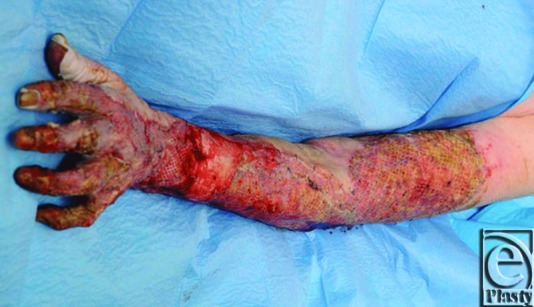
The left forearm at 5 days postoperatively.

**Figure 4:**
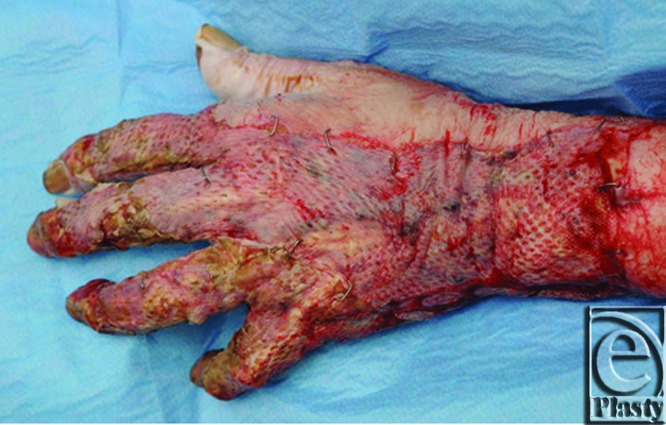
The left hand dorsum at 5 days postoperatively.

Interestingly, the wounds on the arm and hand healed very well with good graft take which did not require regrafting (Figs 3 and 4). She underwent extensive hand therapy and made a good recovery overall despite needing regrafting to the left breast and thigh, the areas where the excision was “fascial” using cutting diathermy. She was nutritionally optimized using nasogastric enteral feeding, which improved her albumin levels to 26 to 30 g/dL. She is now fully medically rehabilitated and has been discharged. At follow-up 6 weeks postoperatively, her wounds were more than 95% healed with no indication of any wound infection.

## DISCUSSION

The WoundWand device uses hydrodissection and radiofrequency ablation technology in one simple, light handheld, hand-operated tool. The probe that interfaces with the tissue is broad-fronted and is well-suited to removing thick eschar from delicate areas such as the dorsum of the fingers and hand.

The device is easy and simple to operate. However, as with all devices there is a learning curve, however in the hands of an experienced burns surgeon, the senior author performed the burn excision with relative ease. A key learning point is to use the device in a unidirectional manner. It was found that repeat back and forth passage obstructs the device-tissue interface, which congests the suction device and causes inefficiency.

The WoundWand device presents itself as a new surgical tool, which is suitable for the discrete tangential excision of full-thickness eschar from delicate areas such as the hand and fingers.
